# Relationship between pretreatment concentration of plasma Epstein‐Barr virus DNA and tumor burden in nasopharyngeal carcinoma: An updated interpretation

**DOI:** 10.1002/cam4.1858

**Published:** 2018-10-30

**Authors:** Liang Peng, Yi Yang, Rui Guo, Yan‐Ping Mao, Cheng Xu, Yu‐Pei Chen, Ying Sun, Jun Ma, Ling‐Long Tang

**Affiliations:** ^1^ Department of Radiation Oncology, State Key Laboratory of Oncology in South China, Collaborative Innovation Centre of Cancer Medicine, Guangdong Key Laboratory of Nasopharyngeal Carcinoma Diagnosis and Therapy Sun Yat‐sen University Cancer Centre Guangzhou China; ^2^ Department of Medical Oncology Guizhou Provincial People’s Hospital Guiyang China

**Keywords:** correlation, liquid biopsy, nasopharyngeal carcinoma, plasma Epstein‐Barr virus DNA, tumor burden

## Abstract

**Background:**

Pretreatment plasma Epstein‐Barr virus (EBV) DNA is an important tumor marker and prognostic factor in nasopharyngeal carcinoma (NPC). This study aimed to clarify the relationship between plasma EBV DNA level and tumor burden.

**Materials and Methods:**

Pretreatment tumor burden was measured by radiologically delineated volumes, including nasopharynx tumor volume (GTVnx) and malignant nodes volume (GTVnd); pretreatment level of plasma EBV DNA was quantified by quantitative polymerase chain reaction. The relationship between natural logarithm of EBV DNA (ln‐DNA) and square root of tumor volume (sq‐GTV) was analyzed by Pearson correlation coefficient and partial correlation coefficient. Correlations in subgroups of tumor and nodal stages were also analyzed. A linear regression model was constructed to evaluate the contribution of tumor volumes to plasma EBV DNA. The prognostic effects of EBV DNA independent of tumor burden were evaluated.

**Results:**

Two thousand two hundred and forty nine nonmetastatic NPC patients with detectable plasma EBV DNA were included in correlation analyses. Ln‐DNA showed significant correlation with sq‐GTVnx (*r* = 0.171) and sq‐GTVnd (*r* = 0.339) separately. Together, sq‐GTVnx and sq‐GTVnd could only explain 12.9% of the ln‐DNA. Tumor and nodal stages of disease could clearly influence the strength of relationship in subgroup analysis. After excluding confounding volume information, EBV DNA still can predict death and distant metastasis, but not locoregional relapse.

**Conclusion:**

This study suggests that plasma EBV DNA is not only an index of tumor burden, but may also reflect other tumor features, such as accessibility to circulation, angiogenesis, tumor cell kinetics, metabolic activity, and metastatic potential, among others.

## 1 INTRODUCTION

Nasopharyngeal carcinoma (NPC) is an endemic disease in Southern China and Southeast Asia. Previous studies^1^, ^2^ revealed that Epstein‐Barr virus (EBV) plays an important role in the pathogenesis of nonkeratinizing subtypes of NPC, which account for more than 95% of the total NPC in endemic areas.^3^ In the past two decades, circulating cell‐free EBV DNA has gained greater recognition in the screening,^4^ prognostication,^5^ and monitoring^6^ of NPC. For NPC patients, it is believed that circulating EBV DNA originates predominantly from the tumor cells rather than other sources (eg, lymphoid tissues), as the EBV genotypes found in matched plasma and tumor samples belong mainly to the same viral clone.^7^ Based on this tenet, the level of circulating EBV DNA is thought to be correlated with tumor burden and to reflect disease stage.^8^

Chan et al^7^ reported a linear relationship between the level of plasma EBV DNA and tumor burden (weight in grams) in a nude mouse model of NPC. Further, Ma et al^9^ validated the hypothesis that pretreatment level of plasma EBV DNA was related to magnetic resonance imaging (MRI)‐delineated tumor volume in 57 locoregionally advanced NPC patients. However, how and to what extent circulating EBV DNA correlates with tumor burden is still poorly demonstrated.

The purpose of this study is to explore the relationship between the level of pretreatment plasma EBV DNA and tumor burden (measured by radiologically determined tumor volume), with the expectation of providing new insights into the significance of circulating EBV DNA and to help better interpret this tumor marker.

## 2 MATERIALS AND METHODS

### 2.1 Pretreatment plasma EBV DNA and tumor volume measurement

As described in previous studies,^10^, ^11^ plasma EBV DNA concentrations were measured by quantitative polymerase chain reaction amplifying the BamHI‐W region of the EBV genome before treatment, and results were expressed as the number of copies of EBV genome per milliliter of plasma.

Before treatment, patients received a computed tomography (CT) simulation scan (Plus 4, Siemens, Erlangen, Germany), including plain and enhanced CT, which extended from the top of the head to 2 cm below the sternoclavicular joint, with 3 mm slice thickness. Usually, an MR simulation scan was also conducted to improve soft‐tissue resolution. Based on the previously reported institutional treatment protocol,^12^ contouring of the target region was performed on an intensity modulated radiotherapy (IMRT) planning system (NOMOS, Pittsburgh, PA) with reference to diagnostic MRI or positron emission tomography and computed tomography (PET/CT). Treatment plans were reviewed and approved by at least three radiation oncologists. Gross tumor volume of nasopharynx lesion (GTVnx) and gross tumor volume of malignant lymph nodes (GTVnd) were calculated by the IMRT planning system. Volume of malignant retropharyngeal lymph nodes was incorporated in GTVnx as they were too close to the nasopharynx lesion to separate. In our study, regional lymph nodes with a shortest axial diameter of 11 mm in the jugulodigastric region, 5 mm in the retropharyngeal region, and 10 mm in all other regions of the neck and a group of three or more lymph nodes with a borderline size were considered malignant. In addition, nodes with necrosis or extracapsular spread were also considered malignant irrespective of size.

### 2.2 Patients

We retrospectively reviewed an inpatient database that included 10 126 patients with newly diagnosed, biopsy‐proven, nonmetastatic NPC treated at Sun Yat‐sen University Cancer Center between April 2009 and December 2015. Patients who did not receive induction chemotherapy before IMRT were included, considering that CT simulation scan was performed after induction chemotherapy and tumor volume would change. Patients without pretreatment plasma EBV DNA data were excluded. Patients whose useful tumor volume data could not be extracted from the IMRT planning system were also excluded. All patients were restaged according to the 8th edition of the American Joint Committee on Cancer (AJCC) staging system based on imaging materials and medical records. The clinical research ethics committee of Sun Yat‐sen University Cancer Center approved this study. As this was a retrospective analysis of routine data, we were granted a waiver of written consent. We have uploaded the essential raw data onto the Research Data Deposit (RDD) public platform (https://www.researchdata.org.cn), with the RDD approval number as RDDA2018000785.

### 2.3 Treatment and follow‐up

Patients were treated with IMRT with or without concurrent chemotherapy. Details of the radiotherapy techniques were reported in a previous study,^12^ with prescribed doses of 66‐72 Gy to the planning target volume (PTV) of GTVnx, and 64‐70 Gy to the PTV of GTVnd. Concurrent chemotherapy was mainly single cisplatin regimen, with 80‐100 mg/m^2^ every three weeks for 2‐3 cycles or 30‐40 mg/m^2^ every week for 5‐7 cycles during radiotherapy.

After treatment was completed, patients were examined at least every 3 months during the first 2 years, then every 6 months for at least 3 years, and annually thereafter or until death. Overall survival (OS) was defined as the time from the initiation of therapy to death from any cause; disease‐free survival (DFS) was defined as the time from initiation of therapy to treatment failure or death from any cause, whichever occurred first; distant metastasis‐free survival (DMFS) was defined as the time from initiation of therapy to first distant failure; locoregional recurrence‐free survival (LRFS) was defined as the time from initiation of therapy to first locoregional failure.

### 2.4 Statistical analysis

To transform the raw data into a more symmetric and normal distribution, we performed natural logarithm transformation of positive EBV DNA (denoted as ln‐DNA) and square‐root transformation of GTV (denoted as sq‐GTV). The primary objective of this study was to investigate the relationship between pretreatment plasma EBV DNA and tumor volume delineated by IMRT planning system, and the strength of this relationship was evaluated by Pearson correlation and partial correlation coefficients. The effects of tumor (T) and nodal (N) categories on the relationship were tested by subgroup analyses. Ln‐DNA was treated as the dependent variable while sq‐GTVnx and sq‐GTVnd were treated as independent variables; the relationship was also evaluated by multivariate linear regression model. The prognostic effect of the residual of ln‐DNA calculated from the linear regression model was explored. In survival analyses, Kaplan‐Meier method, log‐rank test, and multivariate Cox regression model were used. SPSS version 22.0 (IBM Corporation, Armonk, NY, USA) was used for all statistical analyses. Two‐tailed *P*‐values <0.05 were considered statistically significant.

## 3 RESULTS

### 3.1 Patient characteristics

There were 3794 patients meeting the inclusion criteria, of which 1545 patients were negative for plasma EBV DNA and 2249 were positive. Figure S1 shows the selection of patients. Clinicopathological characteristics of included patients are presented in Table 1. Compared to patients with undetectable plasma EBV DNA, patients with positive EBV DNA were characterized by an older age distribution, more smokers, less WHO pathology type I/II NPC, more advanced stage distribution, larger GTVnx and GTVnd, and higher level of pretreatment serum lactate dehydrogenase (LDH). After a median follow‐up of 44 months, the 3‐year OS, DFS, DMFS, and LRFS rates were 93.4%, 86.0%, 91.2%, and 94.2% respectively for the total 3794 patients.

**Table 1:** Clinicopathological characteristics of included patients

Characteristics	Patients with positive EBV DNA (2249 patients)	Patients with negative EBV DNA (1545 patients)	*P*‐value
Age (y)			
Median	46	45	0.015^a^
Range	8‐82	15‐79	
Sex			
Male	1609 (71.5%)	1108 (71.7%)	0.908^b^
Female	640 (28.5%)	437 (28.3%)	
Smoking			
Yes	774 (34.4%)	477 (30.9%)	0.023^b^
No	1475 (65.6%)	1068 (69.1%)	
WHO pathology type			
I	9 (0.4%)	12 (0.8%)	0.030^b^
II	36 (1.6%)	40 (2.6%)	
III	2204 (98.0%)	1493 (96.6%)	
T category^c^			
T1	350 (15.6%)	542 (35.1%)	<0.001^d^
T2	420 (18.7%)	307 (19.9%)	
T3	1126 (50.1%)	605 (39.2%)	
T4	353 (15.7%)	91 (5.9%)	
N category^c^			
N0	274 (12.2%)	682 (44.1%)	<0.001^d^
N1	1278 (56.8%)	713 (46.1%)	
N2	480 (21.3%)	126 (8.2%)	
N3	217 (9.6%)	24 (1.6%)	
Overall stage^c^			
I	72 (3.2%)	329 (21.3%)	<0.001^d^
II	479 (21.3%)	452 (29.3%)	
III	1160 (51.6%)	653 (42.3%)	
IVa	538 (23.9%)	111 (7.2%)	
GTVnx (cm^3^)			
Median	39.24	23.82	<0.001^a^
Range	0.50‐236.47	1.16‐225.07	
GTVnd (cm^3^)			
Median	14.85	1.69	<0.001^a^
Range	0‐264.78	0‐69.46	
LDH (IU/L)			
Median	174.2	169.7	<0.001^a^
Range	67‐1190	73‐1721	
Concurrent chemotherapy			
Yes	1977 (87.9%)	1079 (69.8%)	<0.001^b^
No	272 (12.1%)	466 (30.2%)	

EBV, Epstein‐Barr virus; GTVnx, gross tumor volume of nasopharynx lesion; GTVnd, gross tumor volume of malignant lymph nodes; LDH, lactate dehydrogenase.

a *P*‐value calculated by Mann‐Whitney *U* test.

b *P*‐value calculated by Pearson χ^2^ test.

c According to the 8th edition of the AJCC staging system.

d *P*‐value calculated by Kendall's tau‐b test.

### 3.2 Correlations between EBV DNA and tumor volume

Two thousand two hundred and forty nine patients with positive plasma EBV DNA were included in the correlation analyses. After transformation, means ± standard deviations of ln‐DNA, sq‐GTVnx, and sq‐GTVnd were 8.43 ± 2.20, 6.62 ± 2.26, and 3.86 ± 2.46, respectively. The ln‐DNA was found to be correlated with sq‐GTVnx (*r* = 0.171) and sq‐GTVnd (*r* = 0.339). We also found that sq‐GTVnx was correlated with sq‐GTVnd (*r* = 0.159). The association is statistically significant and the respective confidence intervals are outlined in Table 2.

**Table 2:** Correlation among ln‐DNA, sq‐GTVnx, and sq‐GTVnd for patients with positive EBV DNA

Variables	Pearson correlation coefficient (*r*)	95% Confidence interval	*P*‐value
ln‐DNA vs sq‐GTVnx	0.171	0.128‐0.209	<0.001
ln‐DNA vs sq‐GTVnd	0.339	0.297‐0.377	<0.001
sq‐GTVnx vs sq‐GTVnd	0.159	0.114‐0.200	<0.001

EBV, Epstein‐Barr virus; ln‐DNA, natural logarithm of pretreatment plasma EBV DNA; sq‐GTVnx, square‐root of gross tumor volume of nasopharynx lesion; sq‐GTVnd, square‐root of gross tumor volume of malignant lymph nodes.

**Figure 1:**
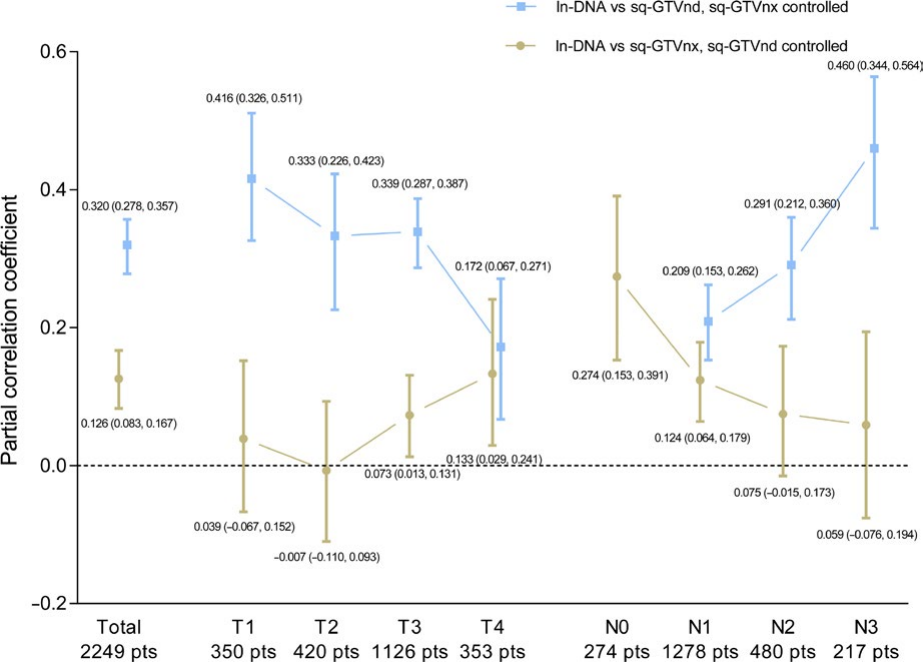
Partial correlation coefficients and 95% confidence intervals for total and subgroup patients with detectable plasma Epstein‐Barr virus DNA. pts, patients

To avoid the influence of association between sq‐GTVnx and sq‐GTVnd, partial correlations between ln‐DNA and sq‐GTVnx (sq‐GTVnd controlled) and between ln‐DNA and sq‐GTVnd (sq‐GTVnx controlled) were analyzed. In addition, subgroup analyses based on T and N categories were conducted to explore the effects of disease stage on the correlations between EBV DNA and tumor volume. Partial correlation coefficients and confidence intervals are presented in Figure 1. For the total patients with positive EBV DNA, sq‐GTVnx and sq‐GTVnd were both independently positively correlated to ln‐DNA. The correlation between sq‐GTVnd and ln‐DNA was stronger than that between sq‐GTVnd and ln‐DNA. For disease in early T category (T1 and T2), the correlation between sq‐GTVnx and ln‐DNA was not statistically significant. From N1 to N3, the correlation between sq‐GTVnd and ln‐DNA became gradually stronger and the two partial correlation coefficients appeared to show an inverse relationship, as one increased and the other decreased.

### 3.3 Linear regression

For patients with positive plasma EBV DNA, ln‐DNA was regressed on sq‐GTVnx and sq‐GTVnd in a multivariate linear regression model (Table 3). The coefficient of determination (*R^2^*) of the regression model was 0.129, which means that 12.9% of the variation in ln‐DNA could be explained by sq‐GTVnx together with sq‐GTVnd. The residual, which is the difference between observed ln‐DNA and ln‐DNA estimated by the regression model, can be interpreted as unexplained variation in ln‐DNA that has nothing to do with sq‐GTVnx and sq‐GTVnd. Histogram and P‐P plot (Figure S2) revealed the residual's normality, and the mean of the residual was 0.

**Table 3:** Multivariate linear regression model for patients with positive EBV DNA (ln‐DNA as dependent variable)

Independent variable	Unstandardized regression coefficient	95% Confidence interval	*P*‐value
sq‐GTVnx	0.117	0.079‐0.155	<0.001
sq‐GTVnd	0.286	0.251‐0.321	<0.001
constant	6.551	6.271‐6.830	<0.001

Abbreviations as in Table 2.

**Figure 2:**
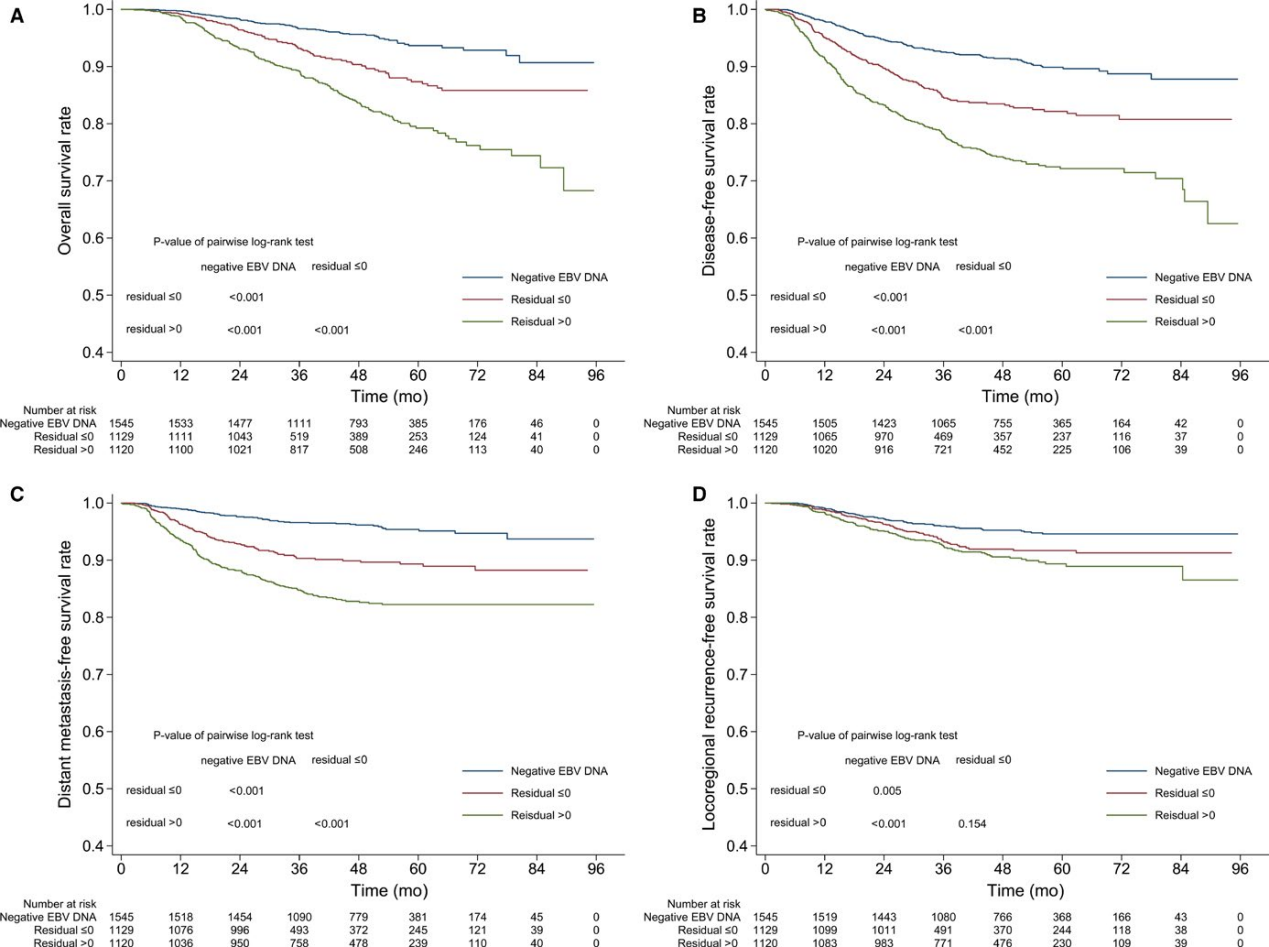
Kaplan‐Meier survival curves based on pretreatment EBV DNA for overall survival (A), disease‐free survival (B), distant metastasis‐free survival (C), and locoregional recurrence‐free survival (D) rates of the whole cohort. Residual is only for patients with detectable EBV DNA and calculated based on the linear regression model presented in Table 3. EBV, Epstein‐Barr virus

Further, the prognostic effects of residual and negative pretreatment plasma EBV DNA were evaluated. All 3794 patients were categorized into three groups: 1545 patients with negative EBV DNA, 1129 with residual ≤0, and 1120 with residual >0. Univariate analyses (Figure 2) showed that negative EBV DNA was a favorable prognostic factor compared to positive EBV DNA in terms of OS, DFS, DFS, and LRFS; residual of ln‐DNA could further predict OS, DFS, and DMFS for patients with positive EBV DNA. Multivariate Cox analyses (Table 4) showed that pretreatment EBV DNA (trichotomized by the negative and residual) was an independent prognostic factor in terms of OS, DFS, and DMFS, but not LRFS.

**Table 4:** Multivariate Cox regression analyses

Outcome	Variables in the final model	HR (95% CI)	*P*‐value
OS	EBV DNA^a^		<0.001
	Negative	Reference	Reference
	Residual ≤0	1.33 (0.97‐1.83)	0.081
	Residual >0	2.15 (1.61‐2.87)	<0.001
	T category		<0.001
	T1	Reference	Reference
	T2	1.34 (0.87‐2.08)	0.181
	T3	1.69 (1.14‐2.52)	0.009
	T4	3.19 (2.04‐4.99)	<0.001
	N category		<0.001
	N0	Reference	Reference
	N1	1.59 (1.11‐2.27)	0.012
	N2	2.79 (1.88‐4.14)	<0.001
	N3	3.82 (2.46‐5.96)	<0.001
	GTVnx (>31.67 vs ≤31.67)	1.56 (1.18‐2.05)	0.002
	Age (>46 vs ≤46)	1.86 (1.49‐2.32)	<0.001
	WHO pathology type (I/II vs III)	2.06 (1.28‐3.33)	0.003
	LDH (>172 vs ≤172)	1.29 (1.03‐1.60)	0.024
	Concurrent chemotherapy (Yes vs No)	0.63 (0.47‐0.83)	0.001
DFS	EBV DNA^a^		<0.001
	Negative	Reference	Reference
	Residual ≤0	1.27 (1.00‐1.62)	0.051
	Residual >0	1.91 (1.53‐2.39)	<0.001
	T category		<0.001
	T1	Reference	Reference
	T2	1.65 (1.20‐2.27)	0.002
	T3	1.78 (1.31‐2.41)	<0.001
	T4	2.83 (1.98‐4.04)	<0.001
	N category		<0.001
	N0	Reference	Reference
	N1	1.38 (1.02‐1.86)	0.038
	N2	2.12 (1.48‐3.02)	<0.001
	N3	2.86 (1.93‐4.23)	<0.001
	GTVnx (>31.67 vs ≤31.67)	1.30 (1.06‐1.61)	0.012
	GTVnd (>8.21 vs ≤8.21)	1.23 (0.99‐1.54)	0.068
	Age (>46 vs ≤46)	1.30 (1.10‐1.54)	0.002
	Smoking (Yes vs No)	1.24 (1.05‐1.47)	0.012
	Cranial nerve invasion (Yes vs No)	1.42 (0.97‐2.09)	0.070
	WHO pathology type (I/II vs III)	2.13 (1.45‐3.15)	<0.001
	LDH (>172 vs ≤172)	1.18 (1.00‐1.39)	0.055
	Concurrent chemotherapy (Yes vs No)	0.71 (0.56‐0.90)	0.005
DMFS	EBV DNA^a^		<0.001
	Negative	Reference	Reference
	Residual ≤0	1.71 (1.22‐2.37)	0.002
	Residual >0	2.60 (1.91‐3.54)	<0.001
	T category		0.001
	T1	Reference	Reference
	T2	1.25 (0.82‐1.89)	0.301
	T3	1.39 (0.95‐2.04)	0.094
	T4	2.32 (1.48‐3.64)	<0.001
	N category		<0.001
	N0	Reference	Reference
	N1	1.63 (1.11‐2.38)	0.013
	N2	2.79 (1.85‐4.22)	<0.001
	N3	3.98 (2.53‐6.27)	<0.001
	GTVnx (>31.67 vs ≤31.67)	1.42 (1.07‐1.87)	0.014
	Sex (Female vs Male)	0.75 (0.58‐0.97)	0.028
	Cranial nerve invasion (Yes vs No)	1.79 (1.15‐2.80)	0.010
	WHO pathology type (I/II vs III)	2.25 (1.38‐3.69)	0.001
	LDH (>172 vs ≤172)	1.37 (1.10‐1.71)	0.005
	Concurrent chemotherapy (Yes vs No)	0.72 (0.53‐0.98)	0.034
LRFS	T category		<0.001
	T1	Reference	Reference
	T2	3.02 (1.83‐4.98)	<0.001
	T3	2.69 (1.68‐4.31)	<0.001
	T4	4.56 (2.69‐7.71)	<0.001
	N category		0.002
	N0	Reference	Reference
	N1	1.03 (0.66‐1.60)	0.907
	N2	1.65 (0.97‐2.82)	0.067
	N3	2.12 (1.16‐3.85)	0.014
	GTVnd (>8.21 vs ≤8.21)	1.53 (1.07‐2.18)	0.020
	Age (>46 vs ≤46)	1.28 (0.99‐1.66)	0.060
	Smoking (Yes vs No)	1.30 (1.01‐1.69)	0.045
	Concurrent chemotherapy (Yes vs No)	0.69 (0.48‐0.98)	0.039

Variables including EBV DNA (trichotomized by the negative and residual), age (dichotomized by median), LDH (dichotomized by median), GTVnx (dichotomized by median), GTVnd (dichotomized by median), sex, smoking, WHO pathology type, T category, N category, cranial nerve invasion, and concurrent chemotherapy were selected by backward elimination method with removal criterion of 0.1.

CI, confidence interval; EBV, Epstein‐Barr virus; GTVnd, square‐root of gross tumor volume of malignant lymph nodes; GTVnx, gross tumor volume of nasopharynx lesion; HR, hazard ratio; LDH, lactate dehydrogenase.

a EBV DNA was trichotomized by the negative and residual; negative means undetectable plasma EBV DNA; the residual was for patients with positive EBV DNA and was the difference between observed ln‐DNA and ln‐DNA estimated by the linear regression model presented in Table 3.

## 4 DISCUSSION

As an important method of liquid biopsy, circulating tumor DNA (ctDNA) has found applications in many tumors.^13^, ^14^, ^15^ In fact, plasma EBV DNA can also be regarded as ctDNA of NPC considering its origin from the tumor cells. Although plasma EBV DNA derives from the EBV genome, which exists in NPC cells in the form of an episome, it still carries useful information about the tumor because the EBV genome and its products are believed to play an important role in oncogenesis and maintenance of malignancy together with somatic mutations.^16^ Since plasma EBV DNA was first identified, researchers have focused on the clinical values of quantification of circulating EBV DNA. It is believed that the concentration of plasma EBV DNA is associated with tumor burden similar to ctDNA from other types of tumor, leading to the question of whether measuring plasma EBV DNA is merely a complicated way of measuring the tumor burden. Since the biological mechanisms behind the presence of EBV DNA in the circulation remain insufficiently elucidated, the question appears to be highly relevant.

It has been a general notion that apoptosis and necrosis of tumor cells account for the release of ctDNA. However, studies also support a mechanism of active release of ctDNA by living tumor cells which is associated with cell proliferation.^17^ Lo et al^18^ found an initial rise in plasma EBV DNA concentration in NPC patients during the first week of radiotherapy and attributed this phenomenon to cell death; considering the rapid clearance of EBV DNA from the circulation,^19^ they then postulated that the decay of plasma EBV DNA during the later period of radiotherapy could reflect the decrease in tumor cell population. However, we think that a half‐life of 3.8 days for plasma EBV DNA as reported by Lo et al may be too fast to explain the gradual tumor regression during radiotherapy we observe in clinical practice. It seems that the concentration of plasma EBV DNA does not simply reflect tumor burden. Conceivably, the concentration of plasma EBV DNA is determined by the rate of release and clearance. As for the clearance of plasma EBV DNA, it seems to be rapid and the spleen, liver, and kidneys may be responsible for clearance.^19^, ^20^ Therefore, concentration of plasma EBV DNA will display a real‐time picture of release. However, factors which may influence the rate of clearance are poorly understood. Possibly, our observation that the elderly population tends to have a higher positive rate of plasma EBV DNA may be partially due to impaired clearance with age. As for the release of plasma EBV DNA from tumor cells, tumor burden is thought to be a pivotal factor positively correlated with the release process. This is what we focused on in the current study.

There is no gold standard for evaluation of tumor burden, butradiologically (ie, CT and MRI) determined tumor volume offers a fairly good choice.^9^, ^21^ However, some drawbacks of this method should also be taken into consideration. In essence, tumor burden is the total tumor cell population and tumor volume can only represent the tumor burden under the condition that the density of tumor cells of respective lesions (ie, nasopharyngeal and nodal) is homogeneous in different patients. Although circulating tumor cells are not thought to be the main source of circulating EBV DNA,^22^, ^23^ the role of micrometastases in releasing EBV DNA is unknown and the tumor burden contribution of micrometastases is hard to evaluate by current methods. High levels of pretreatment plasma EBV DNA can predict metastasis occurring after treatment,^24^ and two possible explanations for this can be postulated: (a) preexisting micrometastases can and do release considerable EBV DNA and are responsible for the high pretreatment levels observed; or (b) an advanced locoregional tumor with biological aggressiveness and propensity to metastasize is responsible for the high level.

In our study, we confirmed the correlation between pretreatment plasma EBV DNA and tumor volume. However, nodal lesion volume is more correlated with EBV DNA than nasopharyngeal lesion volume, which is in accordance with previous studies^9^, ^25^ and means that nodal volume would be more predictive of plasma EBV DNA. A possible explanation is that GTVnx may be inappropriately exaggerated by including invaded anatomical structures (eg, skull base) while the true tumor cell density may be lowered. Another explanation may be in the possible differences between nasopharyngeal and nodal tumor cells in properties of releasing EBV DNA.

We also found that T and N categories could influence the correlations between plasma EBV DNA andtumor volume. The TNM staging system is based on anatomical structures and can also reflect tumor burden to some extent. In subgroup analyses, if the correlation between EBV DNA and GTVnx is strengthened, indicating that the nasopharyngeal lesion may account for an increasing proportion of the total plasma EBV DNA, the correlation between EBV DNA and GTVnd would decrease as the proportion of plasma EBV DNA attributed to the nodal lesion decreased correspondingly. This may explain the inverse relationship between the two partial correlation coefficients (ie, ln‐DNA vs sq‐GTVnx and ln‐DNA vs sq‐GTVnd). For disease in category T1‐T2, the insignificant relationship between ln‐DNA and sq‐GTVnx may be due to confinement of the primary tumor and less vascular invasion.^26^ For disease in category T3‐T4, the nasopharyngeal lesion becomes more important in releasing EBV DNA as the vascular invasion extends. In T4 disease, which is often accompanied by cavernous sinus invasion, the nasopharyngeal lesion has a similar partial correlation coefficient as the nodal lesion. However, Ma et al^21^ did not find a correlation between plasma EBV DNA and vascular invasion in their study, which may be due to a limited number of participants all with locoregionally advanced disease. In subgroup analyses based on N category, an obvious increase in the partial correlation coefficient between ln‐DNA and sq‐GTVnd was found as the N category advanced, which means that it may be easier for advanced nodal lesions to release EBV DNA into the circulation compared to early nodal lesions. We hypothesize that a higher rate of tumor cell loss associated with larger tumor volume and poorer oxygen supply may account for this phenomenon. Furthermore, higher proliferation rates and favorable tumor angiogenesis associated with advanced N category and larger tumor volume may be another explanation.^27^, ^28^, ^29^

In the multivariate linear regression model, tumor volume can only explain 12.9% of the plasma EBV DNA, which is much less than expected. But this reminds us that information hidden within the plasma EBV DNA reflects more than tumor burden. Although incompletely understood, the level of plasma EBV DNA may vary according to not only tumor burden, but also anatomic proximity to vasculature, and biologic features including tumor cell kinetic parameters and metastatic potential.^30^ Tumor angiogenesis is associated with metastasis and may account for release of EBV DNA into circulation. A preclinical research revealed that EBV infection could promote the production of chemokine (C‐C motif) ligand 5, which increases vascular endothelial growth factor expression and NPC angiogenesis by interacting with the PI3K/AKT and hypoxia‐inducible factor‐1α pathways.^29^ It also has been found that level of plasma EBV DNA is also associated with tumor cell metabolic activity measured by PET/CT and circulating cytokines which may reflect the immune and inflammatory status of the body.^25^, ^31^ We postulate that EBV genome copy number in tumor cells may be another factor influencing the plasma EBV DNA.^32^ Previous studies had also noticed the prognostic utility of plasma EBV DNA independent of tumor volume.^33^, ^34^ In our study, we explored the prognostic value of plasma EBV DNA without confounding effects from tumor volume. As expected, plasma EBV DNA without tumor volume information is still an important predictor for OS, DFS, and DMFS, but not LRFS. In the era of IMRT, it seems that the tumor volume is a more useful prognostic factor than plasma EBV DNA in predicting locoregional relapse.

One limitation in our study is the low detection rate (59%) of plasma EBV DNA in the included cohort of untreated NPC patients. This may be a reflection that patients included in this study who did not receive induction chemotherapy were more likely to be in early stage. For the total patients in our database, the detection rate reached 70%, which is still lower than results reported by other institutions.^35^ We postulate that patients with apparent negative plasma EBV DNA were probably not true negatives, but may have had levels too low to be detected by the sensitivity of the methods used by our institution. Considering this possibility, we excluded patients with undetectable plasma EBV DNA in the correlation analyses to avoid bias.

To the best of our knowledge, our study represents the largest population to quantify the relationship between pretreatment plasma EBV DNA and tumor volume. Furthermore, we found the effects of tumor and nodal stage on this relationship. The results of our study demonstrate that level of plasma EBV DNA of NPC patients is not only an index of tumor burden (measured by tumor volume) as has been typically thought, but also an indicator of other tumor features, such as accessibility to circulation, angiogenesis, tumor cell kinetics, metabolic activity, metastatic potential, and other factors. We believe that our study will help clinicians understand the clinical implications of plasma EBV DNA levels in NPC patients in a comprehensive way and make full use of this tumor marker.

## CONFLICT OF INTEREST

None declared.
