# Supplementary figures and images for: Relationship between pretreatment concentration of plasma Epstein‐Barr virus DNA and tumor burden in nasopharyngeal carcinoma: An updated interpretation
Source: Cancer Med. 2018 Oct 30;7(12):5988–98. doi: 10.1002/cam4.1858 (PMC6308091; doi:10.1002/cam4.1858)

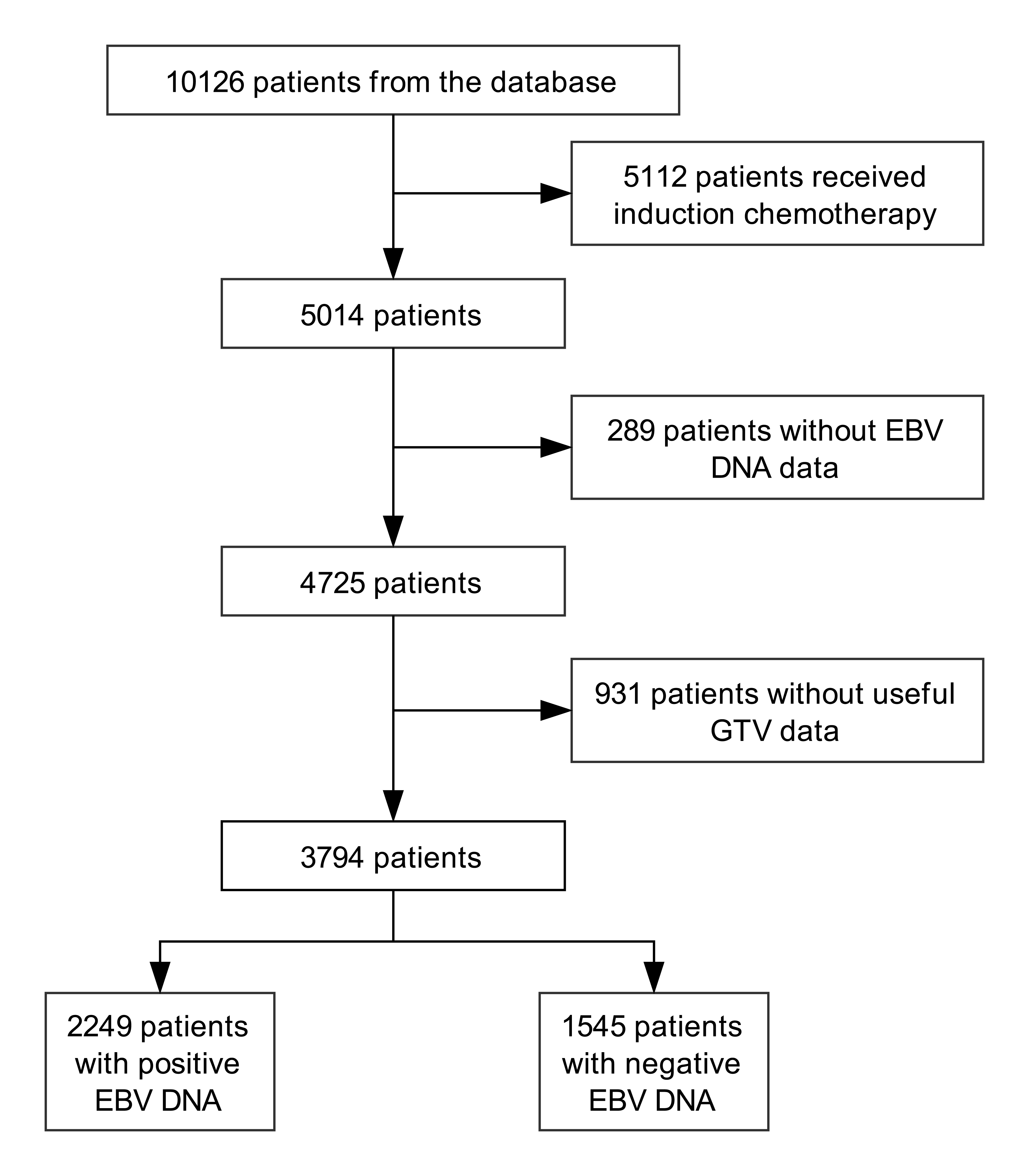

Supplement: Supplementary file 1 [file CAM4-7-5988-s001.tif]

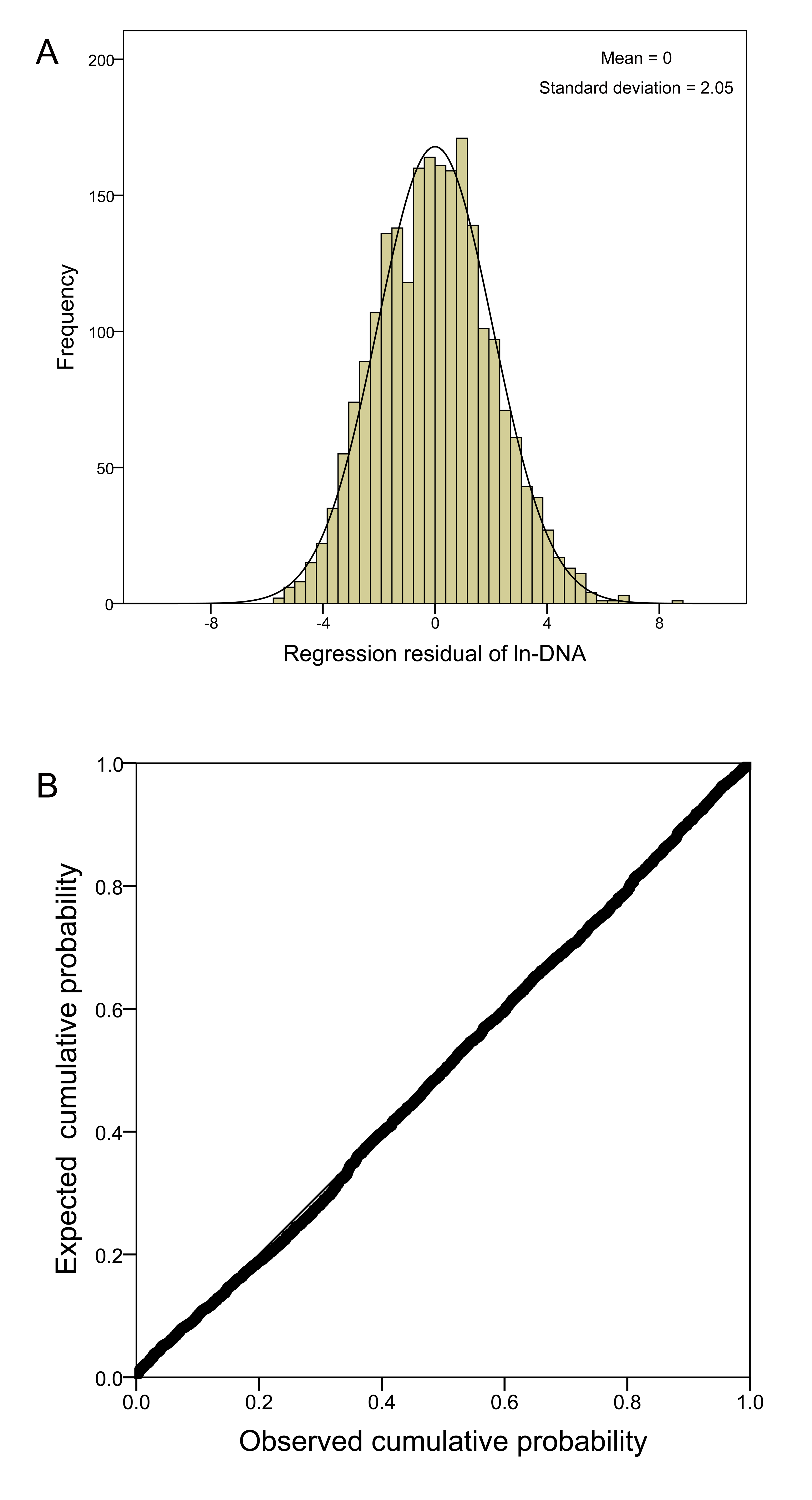

Supplement: Supplementary file 2 [file CAM4-7-5988-s002.tif]
